# Metastatic Medullary Thyroid Carcinoma Without Identifiable Primary Tumor Within the Thyroid Gland, Presenting with Initial Lymph Node Metastasis Followed by Distant Peritoneal Metastasis: A Case Report of a Rare Phenomenon

**DOI:** 10.3390/jcm15072733

**Published:** 2026-04-04

**Authors:** Eunyeong Lee, Jungsup Byun, Moonsik Kim, Jae-Hui Kim, Ji-Young Park, Jongmin Park, An Na Seo

**Affiliations:** 1 Department of Pathology, School of Medicine, Kyungpook National University, Daegu 41405, Republic of Korea; roens1234@naver.com (E.L.); drbyunjs@gmail.com (J.B.); teiroa83@knuh.kr (M.K.); tdc00131@naver.com (J.-H.K.); jyppark@gmail.com (J.-Y.P.); 2 Department of Pathology, Daegu Fatima Hospital, Daegu 41199, Republic of Korea; 3 Department of Radiology, School of Medicine, Kyungpook National University, Daegu 41405, Republic of Korea

**Keywords:** thyroid gland, medullary thyroid carcinoma, peritoneum, calcitonin, *HRAS* mutation

## Abstract

**Background:** We report a rare case of a metastatic neoplasm in the regional lymph nodes and peritoneum whose histopathologic and immunophenotypic profiles were most consistent with a diagnosis of medullary thyroid carcinoma (MTC), although a primary tumor was not histologically demonstrated in the thyroidectomy specimen. **Case presentation:** A 64-year-old man presented with abdominal pain and was found to have increased calcitonin level and a 20 mm lesion in the peritoneum. Peritoneum biopsy revealed plasmacytoid tumor cells which were positive for calcitonin and synaptophysin staining. The patient had a past history of neck dissection due to left side neck mass. The histology revealed metastatic carcinoma with a nested pattern surrounded by fibrous stroma with stromal amyloid deposition. With immunohistochemistry, the findings were most consistent with metastatic MTC, but following total thyroidectomy showed no malignancy. Next-generation sequencing identified a pathogenic *HRAS* mutation, but *RET* mutation was not identified. Despite vandetanib treatment, the disease progressed and the patient expired. **Conclusions:** This case highlights a rare presentation of a metastatic neoplasm highly suggestive of *RET* wild-type MTC with peritoneal involvement, despite the absence of an identifiable primary lesion.

## 1. Introduction

MTC is a malignant neuroendocrine neoplasm originating from parafollicular C cells of the thyroid gland. MTC can secrete calcitonin, which is used for diagnosis and postoperative follow-up [1]. MTC is a rare form of thyroid cancer, accounting for approximately 0.6% of all cases in Korea and 1% to 2% in the United States [1,2,3]. At the time of diagnosis of MTC, 50–75% of patients have lymph node (LN) metastases and 10–15% of patients have distant metastasis [3,4]. The common sites for distant metastases are liver, lung, bone, and mediastinum [5]. Approximately 75% MTC cases present as sporadic form and 25% are as hereditary form, which is associated with multiple endocrine neoplasia (MEN) type 2 [1,3]. *RET* mutations are detected in all patients with inherited MTC and in 40% to 60% patients of apparent sporadic MTC [1,3,6]. Because of the response for targeted *RET* inhibitors, genetic testing for *RET* proto-oncogene mutations is recommended for all patients, regardless of family history [3]. In addition, *RET* mutations are a significant prognostic factor in MTC. Notably, in sporadic tumors, their presence indicates a higher disease stage at diagnosis, increased risk of recurrence, and decreased survival [7]. Herein, we report a rare case of a metastatic neoplasm highly consistent with MTC, presenting with initial regional LN involvement followed by distant peritoneal dissemination, despite the absence of a histologically identifiable primary tumor within the thyroid gland and the presence of wild-type *RET*.

## 2. Case

**Figure 1:**
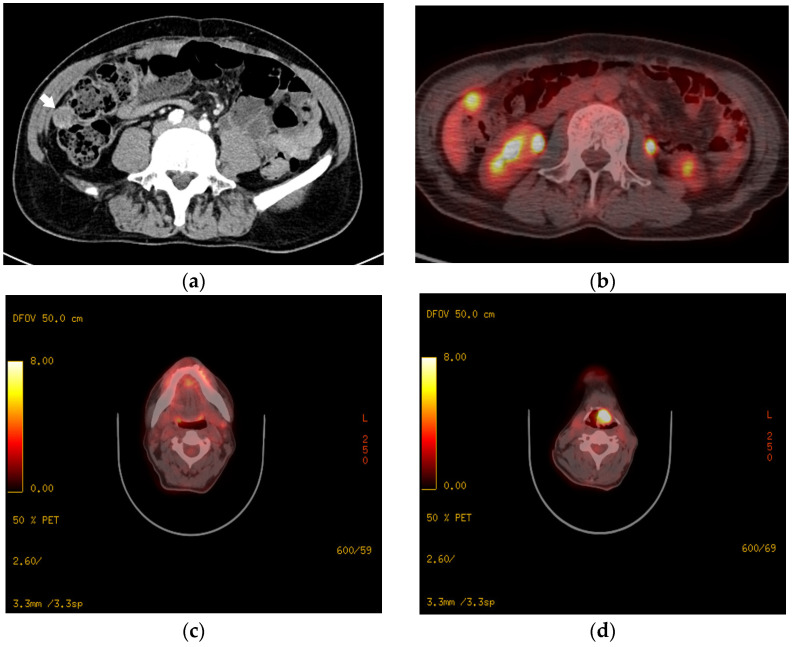
(**a**) Computed tomography (CT) of the abdomen and pelvis revealed an approximately 20 mm lesion in the right lower quadrant (white arrow). On positron emission tomography-CT, hypermetabolic lesions were seen in (**b**) the subhepatic area, (**c**) the left submandibular region, and (**d**) the epiglottis.

A 64-year-old man visited our hospital’s emergency room in December 2023 because of right lower quadrant (RLQ) pain. Abdominal pain started 2 weeks ago, and the symptom worsened when he coughed. On enhanced computed tomography (CT) of the abdomen and pelvis, an approximately 20 mm enhancing lesion in the RLQ, suggestive of peritoneal seeding, was observed (Figure 1a). On positron emission tomography-CT (PET-CT), hypermetabolic lesions were seen in the subhepatic region, left submandibular region, and epiglottis, suggesting malignancy (Figure 1b–d). Abnormal laboratory investigations revealed significantly increased levels of procalcitonin (11.71 ng/mL) and calcitonin (100.54 pg/mL), but a slightly decreased level of calcium (8.4 mg/dL).

**Figure 2:**
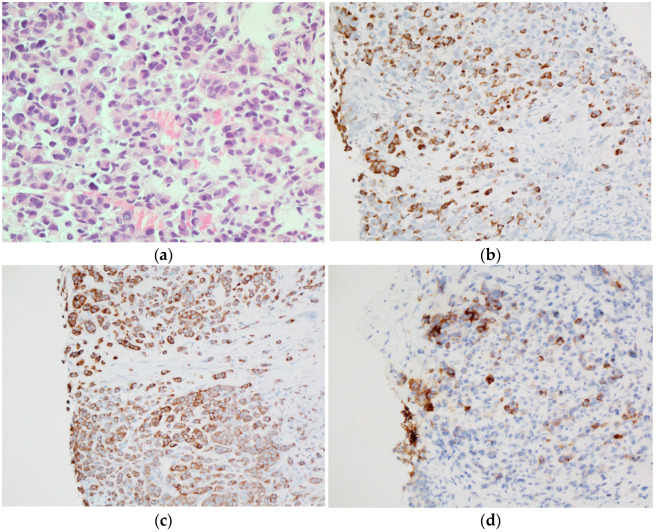
Representative pictures of peritoneal biopsy. (**a**) Scattered plasmacytoid cells with eosinophilic to amphophilic granular cytoplasm are observed (hematoxylin and eosin, 400× magnification). Tumor cells show diffuse immunohistochemistry (IHC) positivity for (**b**) calcitonin and (**c**) synaptophysin (**d**) and focal positivity for CEA (200× magnification).

To evaluate the peritoneal nodule, a core needle biopsy was done. Microscopically, scattered plasmacytoid cells with eosinophilic to amphophilic granular cytoplasm were observed (Figure 2). Round nuclei with finely stippled to coarsely clumped chromatin and indistinct nucleoli were commonly found. Mitotic figures were low, with a maximum of 3/10 high power fields observed. To identify primary origin, immunohistochemistry (IHC) was performed. Tumor cells were seen to present diffuse positivity for cytokeratin (CK), CK7, and synaptophysin, and focal positivity for Carcinoembryonic antigen (CEA) and Insulinoma-associated protein 1 (INSM1), whereas they were negative for CK20, thyroglobulin, P40, GATA3, TTF-1, and PAX-8. Based on the significantly increased serum calcitonin level, calcitonin IHC was performed and strongly positive. Accordingly, the possibility of metastatic MTC or calcitonin-producing neuroendocrine tumor (NET) was considered.

**Figure 3:**
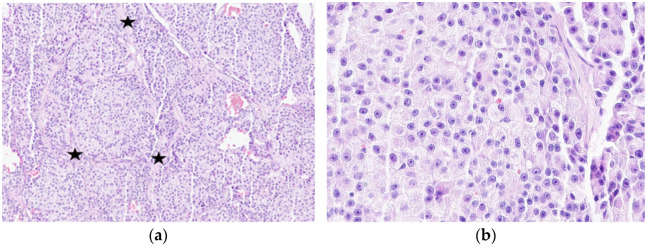

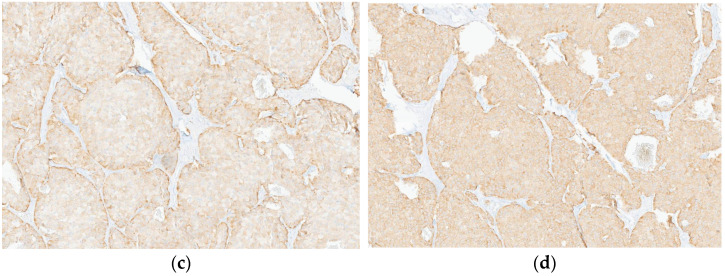
Metastatic lymph node from selective neck dissection. (**a**) Nested pattern was made by fibrous stroma (black star) (hematoxylin and eosin [H&E], 100× magnification). (**b**) Cells were polygonal and plasmacytoid with pinkish granular cytoplasm. In nuclei, there was prominent nucleoli with fine nuclear chromatin. Low-grade atypia was seen. (H&E, 400× magnification). (**c**) Calcitonin and (**d**) synaptophysin immunohistochemistry staining were positive (100× magnification).

According to his past history, in June 2022, he found a 15 mm palpable mass on the left side (level II) of the neck and visited our hospital. Cytologic examination of fine needle aspiration (FNA) of the neck mass revealed high cellularity with singly dispersed plasmacytoid, polygonal, or round tumor cells exhibiting loosely cohesive features and prominent nucleoli. Cytologic diagnosis was considered metastatic carcinoma. In July 2022, PET-CT revealed a hypermetabolic LN at left cervical level II and a focal hypermetabolic lesion in the epiglottis; however, no other sites of uptake suggestive of a primary tumor were identifiable (Supplementary Figure S1). The patient went to another hospital (Asan Medical Center) for a second opinion, where he underwent selective neck dissection (left Ib–V, right II–III), excisional biopsy of epiglottis and tongue, and tonsillectomy. The selective neck dissection revealed metastatic carcinoma in 5 of 26 LNs. Microscopically, metastatic LN showed solid, nested growth patterns separated by fibrovascular stroma with stromal amyloid deposition. Tumor cells were polygonal and plasmacytoid with pinkish granular cytoplasm and prominent nucleoli with fine nuclear chromatin (Figure 3). Immunohistochemically, tumor cells were noted as diffuse positive for calcitonin and synaptophysin, focal positive for CEA, but negative for TTF-1. In contrast, the pathology results of the excised epiglottis revealed an epiglottic cyst. No evidence of malignancy was found in the biopsy specimen of the tongue, nor in the specimens from the bilateral tonsillectomy. Based on these findings, the LNs’ involvement was most consistent with metastatic MTC. Afterwards, he underwent total thyroidectomy in September 2022 at Asan Medical Center. Comprehensive histological evaluation was performed via total embedding of the thyroid gland; however, serial sectioning failed to identify any primary tumor focus. Furthermore, IHC analysis for calcitonin was conducted on representative thyroid blocks, revealing neither a discrete micro-MTC nor evidence of C-cell hyperplasia. Taken together, the diagnosis of this patient was not a calcitonin-producing NET but rather a metastatic MTC with no identifiable primary tumor within the thyroid gland and initially presenting with metastatic LNs. Peripheral blood germline *RET* testing (October 2022, Asan Medical Center) showed no clinically relevant mutations, thereby effectively excluding hereditary MTC and MEN 2. Furthermore, the patient’s medical history was unremarkable for chronic conditions such as hypertension or diabetes, and there was no reported family history of endocrine malignancies or related genetic syndromes. In their hospital, follow-up through June 2023 showed that calcitonin levels remained consistently within the normal range. Normalization of serum calcitonin levels after surgery serves as indirect but strong evidence that the primary cause of hypercalcitoninemia was successfully eliminated. There was no evidence of local tumor recurrence or cervical LN metastasis on ultrasound graphs and no mass-like lesion on laryngoscopy. As mentioned earlier, following an unremarkable postoperative course, the patient returned to our clinic in December 2023 presenting with abdominal pain. In our hospital, next-generation sequencing (NGS) (ONCOaccuPanelTM, NGeneBio, Seoul, Republic of Korea; Illumina NextSeq 550Dx (San Diego, CA, USA), Mid Output Kit v2.5, 150 cycles; Genome version GRCh37/hg19, NGeneAnalysys v1.5) was performed on metastatic LN (tumor content: approximately 70.0%), and *HRAS* missense mutation (p.Gln61Leu, c.182A>T) (variant allele frequency [VAF]: 55.01%) was found as a pathogenic mutation with potential clinical significance, but *RET* mutation was not detected.

The patient started vandetanib treatment in late January 2024. He experienced neck discomfort and abdominal pain. Subsequent CT-enhanced imaging revealed a 9 mm nodular lesion in the right upper quadrant abdominal wall and a lesion in the RLQ. Tumor extension into the epiglottis, vallecula, the anterior and posterior aspects of the epiglottis, and the left aryepiglottic fold, as well as LNsat left levels Ib and II, was also observed. Since February 2024, the patient had been suffering from chronic abdominal pain and had difficulty in swallowing, making it difficult to eat properly. Due to severe general weakness and delirium, the patient was transferred to a hospice ward and died in June 2024.

## 3. Discussion

In our case, the patient presented solely with a left neck mass, which was the only indication for further investigations as he was otherwise asymptomatic. A selective neck dissection was performed to address a potential cancer of unknown primary origin; subsequently, the histopathologic findings, including IHC for calcitonin expression, were found to be most consistent with metastatic MTC. However, a subsequent total thyroidectomy was performed, and MTC was not found in the entire thyroid gland. Although elevated levels of circulating calcitonin are diagnostically significant for MTC, to exclude a primary calcitonin-producing NET, a full work-up was conducted, but no primary source (e.g., pancreas, lung, and larynx) was identified. Assuming primary MTC disappearance, the patient received appropriate treatment. However, two years later, the patient visited the hospital due to abdominal pain, and peritoneal metastases were noted.

Peritoneal metastasis from thyroid cancer is very rare. To our best knowledge, only one case of peritoneal metastasis of MTC has been reported [8]. Aziz, A.L., et al. reported a 38-year-old male patient with MEN2-related MTC who had extrathyroidal extension and positive LNs. He was confirmed to have metastasis in the retroperitoneal nodule several months after undergoing total thyroidectomy and central and lateral LN dissection, followed by radiotherapy [8]. They identified that this patient had a hereditary form of MTC with a L790F mutation in *RET* proto-oncogene, which was found in both blood and retroperitoneal node tests [8]. However, unlike the patient they presented, our case detected an *HRAS* missense mutation (p.Q61L) with VAF of 55.01% as a pathogenic mutation, instead of a *RET* gene mutation on chromosome 10q11.2. This was found using an NGS-based assay on cervical LN from selective neck dissection. Additionally, no clinically relevant mutations were found in the previous peripheral blood germline *RET* mutation test, thereby excluding the possibility of a hereditary syndrome. Furthermore, in our patient, the entire thyroid was examined, but no evidence of malignancy was found. The identification of an *HRAS* mutation of our case provides a critical molecular clue for the diagnosis. A few non-*RET* molecular alterations have been reported in MTC. Nearly 70% of *RET*-wild-type MTCs harbor *RAS* mutations, with *HRAS* being the most common mutation, followed by *KRAS* and *NRAS* [9,10,11].

Al-Angari et al. reported a 34-year-old female patient with metastatic sporadic MTC, noting the absence of primary foci in the thyroid gland [12]. Similar to our case, she had an initial evaluation for a neck mass and a history of a right hemithyroidectomy for a benign cause 20 years before the onset of symptoms. She also underwent FNA of the right cervical LNs, and IHC for calcitonin and synaptophysin was performed on the cell block. At the time of diagnosis, she showed multiple metastases including neck LNs, lung, vertebral bodies, and liver. She underwent completion thyroidectomy with bilateral neck dissection. The thyroid gland revealed a multinodular goiter with no evidence of malignancy, similar to our case. Their patient also showed a wild type of *RET*-proto-oncogene and significantly elevated serum calcitonin.

Serum calcitonin and carcinoembryonic antigen are crucial diagnostic, prognostic, and predictive biomarkers for MTC patients. The European Society for Medical Oncology guidelines recommend 18F-dihydroxyphenyl-alanine-PET/CT for MTC patients with serum calcitonin ≥ 500 pg/mL [13]. For IHC, calcitonin is positive in 95% of MTC cases. Neuroendocrine markers such as synaptophysin, chromogranin A, neuron-specific enolase (NSE) and INSM 1 are also positive. CEA and thyroid-transcription-factor (TTF-1) are also expressed, whereas thyroglobulin is negative in most MTC cases. Notably, calcitonin can also be expressed in various extrathyroid neuroendocrine neoplasms (NENs), especially in intermediate-grade laryngeal NETs [14]. Diffuse and strong positivity for TTF-1 in the MTC may help differentiate it from intermediate-grade laryngeal NETs [14]. Hirsch et al. demonstrated that 1 of 8 (13%) laryngeal moderately differentiated neuroendocrine carcinomas (NECs) showed weak focal positivity for TTF-1, whereas in MTC, 9 of 10 (90%) showed positivity for TTF-1, with seven of these (78%) exhibiting strong diffuse staining [14]. Although our case was negative for TTF-1 in metastatic LNs and peritoneum, no evidence of primary laryngeal cancer was observed in the full work-up. Clinical follow-up further reinforced the diagnosis. Despite comprehensive embedding of the entire thyroid gland failing to reveal a macroscopic primary tumor, the postoperative normalization of serum calcitonin provides compelling indirect evidence that the source of hypercalcitoninemia was successfully eradicated.

Feola, T., et al. [15] reported a 59-year-old male patient with dysphagia, dyspnea, and a left lateral cervical mass who presented with an increasing serum calcitonin level (50 pg/mL). Their case revealed a lesion of the supraglottic left hemilarynx, and it was biopsied and diagnosed as a moderately differentiated NET [15]. The total thyroidectomy specimen revealed benign hyperplastic nodules in the thyroid gland [15]. A cervical LN was diagnosed as metastatic NET due to positivity for chromogranin A and synaptophysin, although calcitonin and TTF-1 showed strong positivity, unlike our case [15].

Interestingly, Xu, B., et al. reported seven cases of metastatic papillary thyroid carcinoma without an identifiable primary tumor despite extensive microscopic examination of the entire thyroid gland [16]. They hypothesized that the primary tumor was not found because microcarcinoma smaller than the thickness of the tissue block might be missed, and tumor regression might have occurred [16]. Because tumor size of MTC ranges from less than 1 mm in diameter to those that replace an entire thyroid lobe, we also agree with their opinion regarding MTC.

Our case illustrates a rare phenomenon involving a suspected MTC without a demonstrable primary lesion even after an exhaustive pathologic examination. This adds to the interesting spectrum of metastatic MTC-like presentations, including unusual sites such as the peritoneum. Moreover, in our case, an *HRAS* mutation was observed, not a *RET* mutation that is associated with inherited form and tumor aggressiveness. This report underscores the diagnostic complexity of occult MTC and the pivotal role of molecular profiling. 

## Data Availability

The datasets generated or analyzed during the study are available from the corresponding author on reasonable request.

## Supplementary Materials

The following supporting information can be downloaded at: https://www.mdpi.com/article/10.3390/jcm15072733/s1, Figure S1: Patient’s ^18^F-FDG positron emission tomography maximum-intensity projection image.

## Abbreviations

The following abbreviations are used in this manuscript:

MTC	Medullary thyroid carcinoma
MEN	Multiple endocrine neoplasia
CT	Computed tomography
PET-CT	Positron emission tomography-computed tomography
IHC	Immunohistochemistry
NET	Neuroendocrine tumor
FNA	Fine needle aspiration
NGS	Next-generation sequencing
VAF	Variant allele frequency
