# Supplementary figures and images for: Metastatic Medullary Thyroid Carcinoma Without Identifiable Primary Tumor Within the Thyroid Gland, Presenting with Initial Lymph Node Metastasis Followed by Distant Peritoneal Metastasis: A Case Report of a Rare Phenomenon
Source: J Clin Med. 2026 Apr 4;15(7):2733. doi: 10.3390/jcm15072733 (PMC13073370; doi:10.3390/jcm15072733)

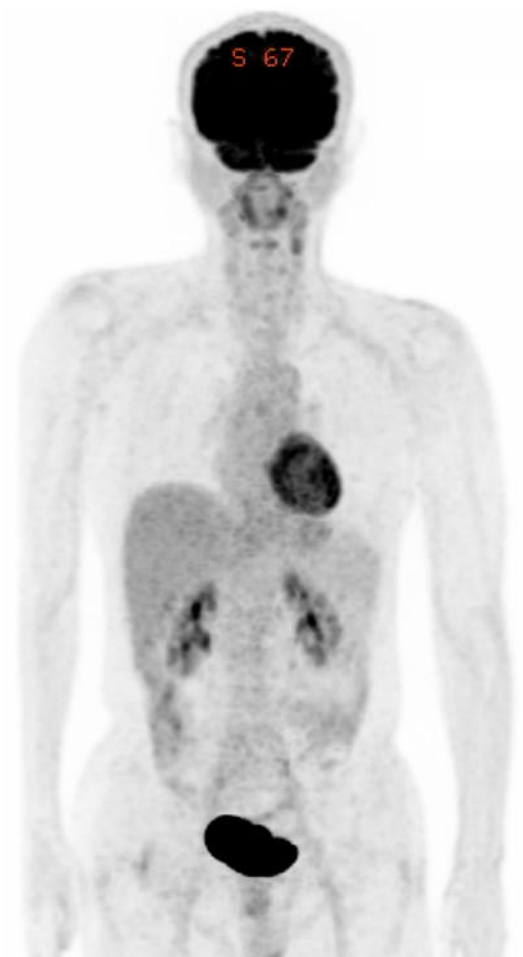

Supplementary Figure S1

Supplement: Supplementary file 1 [file jcm-15-02733-s001.zip › jcm-4188456-supplementary.pdf]
